# Intereleukin-10 Promoter Polymorphism in Mild Cognitive Impairment and in Its Clinical Evolution

**DOI:** 10.4061/2010/854527

**Published:** 2010-07-20

**Authors:** Beatrice Arosio, Luigina Mastronardi, Carlo Vergani, Giorgio Annoni

**Affiliations:** ^1^Department of Internal Medicine, Università degli Studi di Milano, Geriatric Unit, Fondazione IRCCS Cà Granda Ospedale Maggiore Policlinico, Via Pace 9, 20122 Milano, Italy; ^2^Department of Internal Medicine and Prevention, Università degli Studi di Milano-Bicocca, Geriatric Clinic, San Gerardo Hospital, Via Pergolesi 33, 20052 Monza, Italy

## Abstract

Specific proinflammatory alleles are associated with higher risk of Alzheimer disease (AD) in different onset age. The homozygosis for the A allele of −1082 polymorphism (G/A) of interleukin-10 (IL-10) promotes a higher risk of AD and reduced IL-10 generation in peripheral cells after amyloid stimulation. In this paper we analysed genotype and allele frequencies of this polymorphism in 138 subjects with mild cognitive impairment (MCI) diagnosed, respectively, as amnestic (a-MCI) and multiple impaired cognitive domains (mcd-MCI). The genotype frequencies were similar in a-MCI and AD subjects, whereas in mcd-MCI comparable to controls (AA genotype: 50% in a-MCI, 49.2% in AD, 28.7% in mcd-MCI and 31.8% in controls). Consequently, both allele and genotype distributions were significantly different between a-MCI and mcd-MCI (allele: *P* = .02, genotype: *P* < .05). These results support the theory that polymorphisms of cytokine genes can affect neurodegeneration and its clinical progression. IL-10 may partly explain the conversion of a-MCI to AD or be a genetic marker of susceptibility.

## 1. Introduction

The pathogenic process of Alzheimer's disease (AD) starts decades before the clinical onset of the disease [1]. During this preclinical phase, there is a gradual loss of axons and neurons, and at a certain threshold the first symptoms, most often impaired episodic memory, appear. At this stage, patients do not fulfil the criteria for dementia and may be diagnosed with mild cognitive impairment (MCI). There is considerable clinical heterogeneity of this pathology since different clinical patterns can be recognized: amnestic MCI (a-MCI), MCI with multiple impaired cognitive domains (mcd-MCI), and single nonmemory domain MCI [2]. Although a-MCI may be the preclinical stage of AD, there is no established method to predict progression to AD in individuals with MCI.

Inflammation is accepted to be a feature of AD [3, 4] and the pathogeneses of neurodegeneration have been at least in part attributed to the release of proinflammatory cytokines from brain resident cells [5, 6] and, although less consistently, from peripheral cell [7, 8]. Furthermore, an increased intrathecal production of the proinflammatory cytokine TNF-*α* and a decreased production of the anti-inflammatory cytokine TGF-*β* have been demonstrated in the brain of patients with MCI, suggesting there is a proinflammatory state in such patients at high risk for AD [9].

Moreover, circulating acute phase reactant levels in middle age predict AD risk in old age and in particular certain functional promoter polymorphisms in cognate genes that modulate inflammation are often found at elevated frequency among AD cases. 

Recently specific risk sets of proinflammatory alleles were identified that characterize AD in different onset age (before age 65, at ages 65–74, and at older ages) [10].

These alleles comprise also the −1082 promoter gene polymorphisms of IL-10 (G/A substitution) [11].

IL-10 maps to chromosome 1 between 1q31 and 1q32 is highly polymorphic, and its production is correlated to biallelic polymorphisms at positions −1082 (G to A), −819 (T to C), and −592 (A to C). The polymorphism at position −1082 lies within an Ets (E-twenty-six specific)-like recognition site and may affect the binding of this transcriptional factor and, therefore, alter transcription activation; the −1082 A allele correlates with low IL-10 generation after stimulation of T cells in vitro [12], while polymorphisms at positions −819 and −592 do not seem to be involved.

In a previous study, we found that the homozygosis for the A allele of the IL-10 −1082 G/A single nucleotide polymorphism (SNP) was associated with six-fold higher risk of AD. In the same study, we also analysed the production of IL-10 in Peripheral Blood Mononuclear Cells (PBMCs) of AD patients and age-matched controls after specific stimulation with amyloid peptide, LPS, and Flu. Since the generation of IL-10 was reduced in patients after amyloid stimulation, we concluded that these specific immune responses may be selectively impaired in AD [13]. 

The aim of this study was to analyse the genotype and allele frequencies of these IL-10 SNPs in 138 subjects with MCI and to compare them with those previously shown in AD and healthy controls (HCs) [13].

## 2. Materials and Methods

### 2.1. Study Protocol

This study comprised 138 subjects with MCI age 80.37 ± 5.93 years (mean ± standard deviation (SD)). All patients were Caucasian, living in Northern Italy, and selected from a larger ambulatory population sample followed at the Geriatric Unit of the Ospedale Maggiore Policlinico IRCCS, University of Milan, Italy and the Geriatric Clinic of the University of Milan-Bicocca, Italy.

At enrolment, MCI subjects were divided into two groups based on cognitive features and diagnosed, respectively, as a-MCI (30 patients) and mcd-MCI (108 patients).

In particular, a-MCI met the criteria described by Petersen [14]: subjects with memory impairment only (>1.5 SD above the age- and education-specific norms) and no difficulties in any other area of cognitive functions. mcd-MCIs were subjects diagnosed with impairment in at least two cognitive domains of more than 1 SD below the mean of the respective age- and education-matched population, and with cognitive decline confirmed by the individuals themselves or reliable informants, but in whom no diagnosis of dementia could be achieved. A cut-off score of 1 SD was applied, which is less severe than that used for a-MCI, in order to obtain higher diagnostic sensitivity even though diagnostic specificity was reduced. Because the presence of more than one cognitive deficit and frequently initial impairment in Lawton's instrumental activities of daily living also characterized mcd-MCI, it may be mistaken for dementia; thus, a less severe criterion (>1 SD) allows better differentiation between mcd-MCI and dementia [15].

At this time, 74 patients out of the 138 completed a four-year follow-up and 24 were diagnosed with AD, 22 with vascular dementia (MCI→VD), and 28 with stable MCI [16]. Subjects who developed AD during follow-up were required to meet the DSM IV (Diagnostic and Statistical Manual of mental Disorders—4th ed.) and NINCDS-ADRDA (National Institute of Communicative Disorders and Stroke-Alzheimer's Disease and Related Disorders Association Work Group) criteria [17].

Within MCI who progressed to AD (MCI→AD), only two were diagnosed as mcd-MCI at enrolment; all the others were diagnosed as a-MCI.

In order to minimize the risk of possible inflammatory processes, all subjects were selected in the absence of clinical signs of inflammation (e.g., normal body temperature, no concomitant inflammatory condition) and with normal blood chemistry (red blood cell sedimentation rate, albumin, transferring, and C reactive protein plasma levels).

Informed consent was obtained from all subjects and the Ethics Committee of both universities approved the study, which was conducted according to the Helsinki II declaration. This population was matched with AD patients (*n* = 63) and nonsdemented sex- and age-matched healthy controls (*n* = 63) enrolled for our previous study [13].

### 2.2. Gene Polymorphism Analysis

Whole blood was collected by venipuncture in Vacutainer tubes containing EDTA (Becton Dickinson Co., Rutherford, NJ).

Genomic DNA was extracted by the salting-out method as described in [18]. The concentration and purity of DNA were determined by spectrophotometric analysis. In order to establish IL-10 genotypes we employed a polymerase chain reaction using sequence-specific primers (PCR-SSPs). The sequence in the promoter region of the IL-10 gene (polymorphic positions −1082, −819, and −592) was amplified using the cytokine genotyping tray method (One Lambda, Canoga Park, CA, USA). The human *β*-globin gene was amplified as an internal control for the genomic DNA preparation. PCR conditions were indicated by the One Lambda PCR program (OLI-1) and the PCR products were visualised by electrophoresis in 2.5% agarose gel.

ApoE genotypes were determined by means of PCR amplification of a 234 base-pair fragment of exon 4 of the ApoE gene, followed by digestion with Cfo1. The restriction patterns were revealed by means of 4% agarose gel electrophoresis [13].

### 2.3. Statistical Analysis

Statistical analysis was performed with the SPSS statistical package (SPSS version 17, Chicago, IL). Genotype and allele frequencies in the study groups were compared using the *χ* ^2^-test. *P* < .05 was taken as the cut-off for statistical significance.

## 3. Results

### 3.1. Distribution of IL-10 Genotypes in MCI Subjects

The genotype and allele frequencies of the biallelic polymorphism at position −1082 are reported in Table 1. This SNP alters transcriptional activation with a gene dosage-related effect, so GG genotype correlates with high, GA with intermediate, and AA with low IL-10 production after stimulation of T cells in vitro [12]. 

As previously described [13], AD patients show a significant higher frequency of the −1082A low producer allele, which skews the genotype distribution in AD compared to HC, with a significant decrease of −1082 GG high producer genotype. 

MCI subjects as a whole had an intermediate pattern between AD and HC subjects, the percentages of G allele and GG genotype being 40.9% and 15.2%, while the percentage of A allele and AA genotype being 59.1% and 33.3%, respectively, (allele: *P* = .02, genotype: *P* = .05) (Table 1).

It is interesting to note that the genotype frequencies of the −1082 SNP in a-MCI subjects were similar to those of AD subjects, whereas those of mcd-MCI were comparable to HC (AA genotype 50% in a-MCI and 49.2% in AD; 28.7% and 31.8% in mcd-MCI and HC, resp.) (Table 2). Consequently, the allele and genotype distributions were significantly different between a-MCI and mcd-MCI (allele: *P* = .02, genotype: *P* < .05).

The same SNP is linked with two other SNPs at positions −819 and −592. They combine with microsatellite alleles to form haplotypes where the difference in IL-10 production is mainly accounted by the −1082 SNP [19, 20]. The genotype and allele frequencies of −819 and −592 SNPs were distributed similarly in our samples (data not shown).

### 3.2. Distribution of Apolipoprotein E Genotype in MCI Subjects

The frequency of ApoE *ε*4 in our sample was in line with the data already published [21–24]. In particular genotyping of our MCI patients globally considered revealed the presence of *ε*4 allele in 40% of cases and, during follow-up, in 54% of MCI→AD and 39% in stable MCI. The ApoE4 status is an independent risk factor for AD [13].

### 3.3. Follow-Up

After a 4-year follow-up 24 MCI progressed to AD (MCI→AD) [16] and 22 progressed to vascular dementia (MCI→VD). Table 3 shows −1082 SNP distributions in MCI progressing and not progressing to AD (stable MCI).

In MCI→AD both A allele and AA genotype were higher than in stable MCI and in MCI→VD.

Due to the limited number of patients that completed the follow-up period, the data reached the statistical significances only comparing genotype and allele percentage (allele: *P* < .05, genotype *P* = .004).

## 4. Discussion

A “cytokine cycle” has been proposed where [25] the anti-inflammatory cytokines (IL-4, IL-10, and IL-13) regulate *β*-amyloid-induced microglial/macrophage inflammatory responses and modify the microglial activity surrounding amyloid neuritic plaques [26]. These cytokines can inhibit the induction of IL-1, TNF-*α*, and MCP-1 in differentiated human monocytes and, above all, IL-10 causes dose-dependent inhibition of the IL-6 secretion induced by *β*-amyloid in these cells and in murine microglia [25].

In a previous paper, we described not only a significantly higher percentage of IL-10 −1082 AA low-producing genotype among AD cases, but also a reduced IL-10 generation in peripheral blood mononuclear cells from these patients after *β*-amyloid stimulation [13]. 

Interestingly a report on Italian centenarians, who are clearly less prone than younger persons to age-related diseases, showed that extreme longevity is significantly associated with the high IL-10-producing genotypes [27].

In the present study, the allele frequencies of −1082 SNP in a-MCI subjects were similar to those of AD patients, whereas those of mcd-MCI were comparable to HC (the frequencies of the low-producer AA genotype were 50% and 28.7%, in a-MCI and mcd-MCI, resp.).

It is to note that, after an adequate period of follow-up, the twenty-four a-MCI subjects that progressed to AD showed a higher percentage of AA carriers (41.7%) compared to those of MCI that remain stable (28.6%) and compared to those progressed in vascular dementia (27.3%). The similar genotype distribution of this IL-10 SNP in AD and a-MCI but not in mcd-MCI and the data retrospectively obtained after the follow-up suggest that it is potentially involved in the conversion of a-MCI to AD.

However, our results support the theory that the overall risk of developing AD may be governed by a multifactorial “susceptibility profile” and that polymorphisms of cytokine genes can affect neurodegeneration and its clinical progression. 

In addiction, IL-10 may partly explain the conversion of a-MCI to AD or, at least, be a genetic marker of susceptibility [28].

Therefore, it is extremely relevant to closely define intrinsic (i.e., genetic) individual risk profiles in prevention and treatment trials. The finding that the set of gene variants in innate immunity associated with earlier onset predicted rapid clinical progression suggests that interventions to control inflammation might be useful especially for relatively younger cases to delay disease progression.

## Figures and Tables

**Table 1:** Distribution of genotype and allele frequencies of −1082 (G/A) SNP in Alzheimer's disease patients (AD), control subjects (CT), and mild cognitive impairment patients (MCI).

	GG (H)	GA (M)	AA (L)	G	A
AD	4 (6.4%)	28 (44.4%)	31 (49.2%)	36 (28.6%)	90 (71.4%)
CT	14 (22.2%)	29 (46%)	20 (31.8%)	57 (45.2%)	69 (54.8%)
MCI	21 (15.2%)	71 (51.4%)	46 (33.3%)	113 (40.9%)	163 (59.1%)

Genotype: *χ* ^2^ 9.480, d.f. 4; *P* = .05.

Allele: *χ* ^2^ 8.257, d.f. 2; *P* = .02.

**Table 2:** Distribution of genotype and allele frequencies of −1082 (G/A) SNP in amnestic MCI (a-MCI) and multiple cognitive domains MCI patients (mcd-MCI).

	GG (H)	GA (M)	AA (L)	G	A
a-MCI	1 (3.3%)	14 (46.7%)	15 (50%)	16 (26.7%)	44 (73.3%)
mcd-MCI	20 (18.5%)	57 (52.8%)	31 (28.7%)	97 (44.9%)	119 (55.1%)

Genotype: *χ* ^2^ 6.927, d.f. 2; *P* < .05.

Allele: *χ* ^2^ 5.729, d.f. 1; *P* = .02.

**Table 3:** Distribution of genotype and allele frequencies of −1082 (G/A) SNP in MCIs that remain stable, progressed to AD (MCI→AD), and progressed to VD (MCI→VD).

	GG (H)	GA (M)	AA (L)	G	A
MCI stable	8 (28.6%)	12 (42.8%)	8 (28.6%)	28 (50%)	28 (50%)
MCI→AD	2 (8.3%)	12 (50%)	10 (41.7%)	16 (33.3%)	32 (66.7%)
MCI→VD	5 (22.7%)	11 (50%)	6 (27.3%)	21 (47.7%)	23 (52.3%)

Genotype distribution compared percentages: *χ* ^2^ 15.604, d.f. 4; *P* = .004.

Allele distributions compared percentages: *χ* ^2^ 6.661, d.f. 2; *P* < .05.
